# Supplementary material for: Random sequences rapidly evolve into de novo promoters
Source: Nat Commun. 2018 Apr 18;9:1530. doi: 10.1038/s41467-018-04026-w (PMC5906472; doi:10.1038/s41467-018-04026-w)
Supplement: Supplementary file 1 — Supplementary Information [file 41467_2018_4026_MOESM1_ESM.pdf]

Random Sequences Rapidly Evolve into De Novo Promoters

Yona et al.

Supplementary Information

Supplementary Figure 1

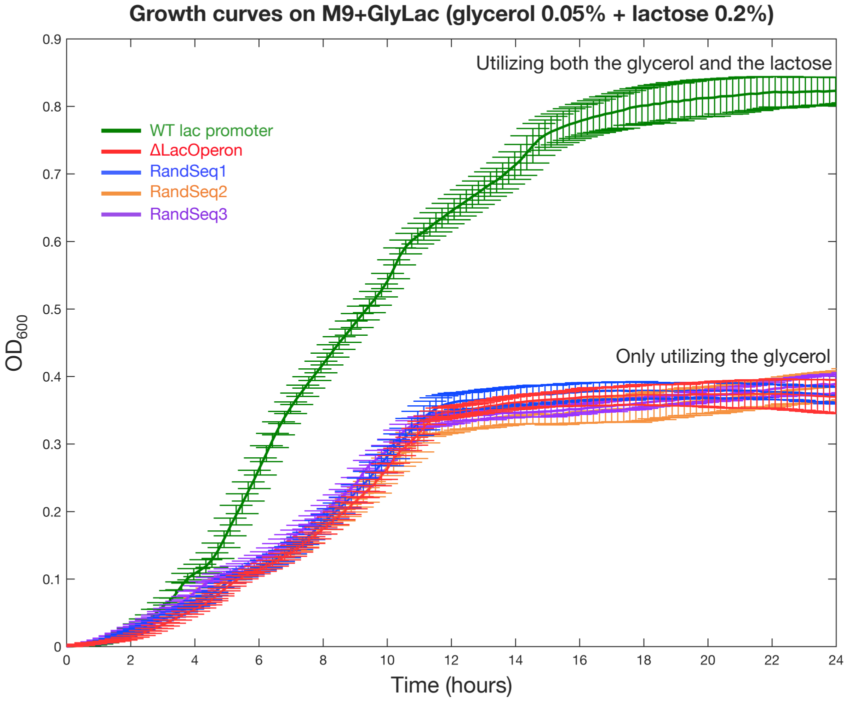

Supplementary Figure 1:
Replacing the WT lac promoter with a random sequence typically abolishes the ability to utilize lactose - Growth curve measurements of WTpromoter (green), ΔLacOperon (red) and RandSequence1, 2, 3 (blue, orange and purple respectively). Shown in values of optical density (OD600) over time during continuous growth on minimal medium (M9+GlyLac, glycerol 0.05% plus lactose 0.2%) at 37°C. The random sequence strains can only utilize the glycerol in the medium and show a growth curve very similar to the ΔLacOperon strain in which the lac genes were deleted. The difference in growth curves between the random sequence strains to WTpromoter reflects the adaptive potential for de novo expression of the lac genes. Error bars represent s.e.m of readings from 16 different wells per strain, in a 96 well plate.

Supplementary Figure 2

Locating the promoter motifs of random sequences that enabled lactose utilization before evolution

Evolution of RandSeq7

```
ctgctttgtactcatggtacgggaaggatcccagattctctagacacacggttgtgatgttgtatatacttgttgcttatggttttccccttcggaagtggcg
                                        TtGACA________18________TATAaT
```

Evolution of RandSeq12

```
ccgcccgattgaagcgaacccggatgatatcgcatgatatgtgaggattagccgatcagtaccaataccgaagagattatgtccttgatcagcaggcaggaga
        TTGAca________18________tATaAT
```

Evolution of RandSeq30

```
gtcggcccggccgtccatcgactctatatcgtatatacttgctttataccaattctcaacctcatatgcttcacgattcaggctactagtgggtgaagttcac
                                          TTgAcA_______16_______TATaaT
```

Evolution of RandSeq34

```
cagtttcattaaccaatggacagtatatatactaaggctatcgtgtgattgggaggagcgcctctctgaactcgtgtgctctttgtctcaccggaacgccttt
    TTgAca_______17________TATAaT
```

Supplementary Figure 2:
Realizing promoter motifs in the random sequences that were already active promoters before evolution - Shown are the sequences of RandSequences7, 12, 30, 34 and the locations of promoter motifs in the random sequences. For these four strains, we observed the ability of cells to grow on lactose-only plates (M9+Lac) without any adaptation. Below each random sequence the canonical promoter is shown where capital bases indicate a match to the canonical motifs TTGACA and TATAAT.

Supplementary Figure 3

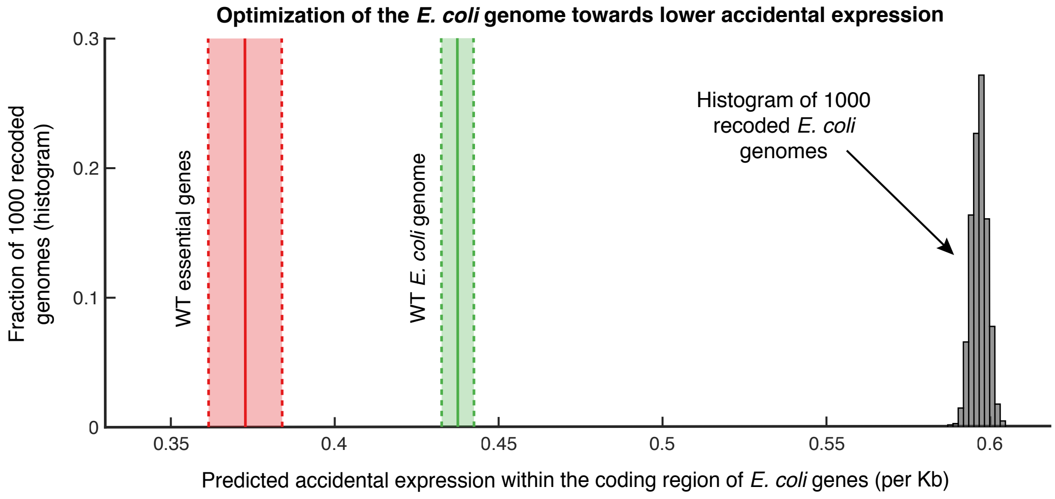

Supplementary Figure 3:
Selection against the occurrence of random promoters in the coding region of genes
We evaluated promoters that accidentally occur across the genome by searching for promoter motifs in the coding region of *E. coli*. As a reference we did the same evaluation for 1000 alternative versions of the *E. coli* coding region by recoding each gene with synonymous codons while preserving the amino acid sequence and the codon bias. Accidental expression of the thousand recoded versions of *E. coli* are shown by a histogram (grey), and the accidental expression of the WT *E. coli* genome is shown by vertical solid lines, for all genes in green, and for the subset of essential genes in red. Shaded areas around the vertical solid lines represent s.e. (delineated by vertical dashed lines). The WT version of the genome is significantly depleted for promoter motifs, indicating genome-wide minimization of accidental expression.

Supplementary Figure 4

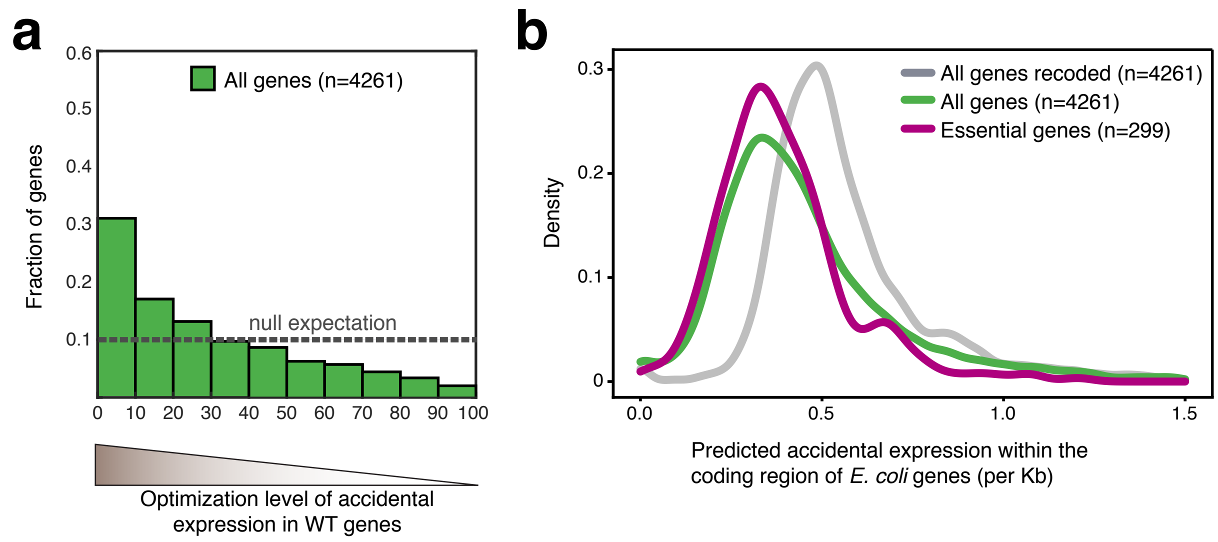

Supplementary Figure 4:
Selection against the occurrence of random promoters in the genome – alternative null model
We evaluated promoters that accidentally occur across the genome by searching for promoter motifs in the coding region of E. coli. As a reference we did the same evaluation for 1000 alternative versions of the E. coli coding region by shuffling the codons of each gene, which maintains the GC content and codon bias of each gene. Comparing the WT genes to the 1000 shuffled versions allowed us to look for codon combinations that might have been under negative selection in the WT genome. For example, the shuffled versions can indicate if a combination of two specific codons is avoided in the WT genes because it creates a promoter motif inside a gene. **(a)** A score for accidental expression is calculated for each WT gene and a rank is assigned to each gene by its order in the scores of its 1000 shuffled versions. Shown is the histogram of ranks (divided into deciles) for all WT genes demonstrating that ~30% of WT genes are ranked at the most optimized decile. Dashed line shows expected histogram if WT genes had similar values to their shuffled versions. **(b)** Density plots of accidental expression in the coding sequences of E. coli genes. Distribution of a thousand shuffled versions of E. coli coding region are shown in grey (the value that represent each gene is the median of its 1000 shuffled versions), the accidental expression of the WT E. coli genes is shown in green, and for the subset of essential genes in magenta. The WT version of the genome is significantly more depleted for promoter motifs, indicating genome-wide minimization of accidental expression. This minimization is further emphasized for the essential genes.

Supplementary Figure 5

a

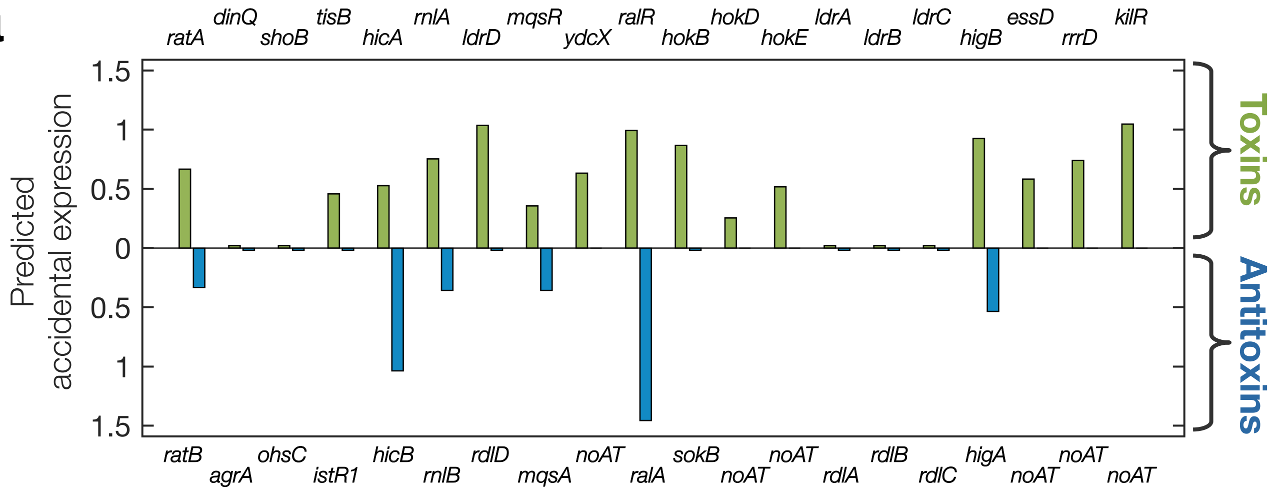

b

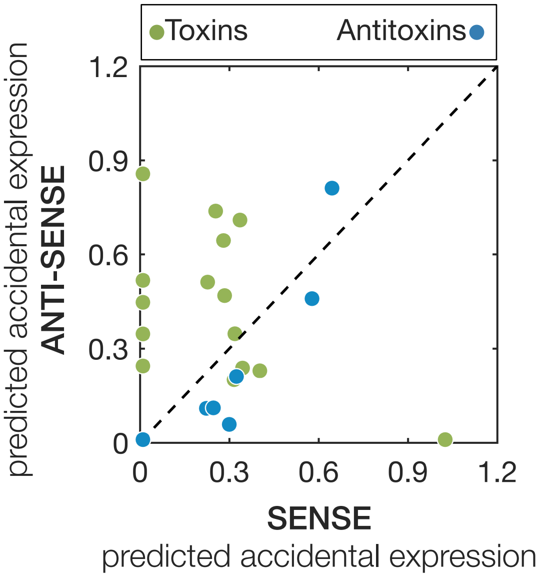

Supplementary Figure 5:

Accidental expression within toxin genes might be selected for as a means to control their expression - For each toxin-antitoxin couple the accidental expression scores examined for differences between toxins genes to their antitoxins and between accidental expressions in 'sense' (with the direction of the gene) compared to the 'antisense' (against the direction of the gene). **(a)** Accidental expression scores are compared between toxins (above the X-axis) and their antitoxin (below the X-axis) showing a tendency of toxins to have higher accidental expression compared with their antitoxin counterparts. **(b)** For both toxin and antitoxin genes the accidental expression was split into 'sense' and 'antisense' direction. While in antitoxin genes the two components tend to correlate (as generally seen in the genome, see Supplementary Fig. 6) in the toxins genes the 'antisense' direction is significantly higher, which may imply that E. coli selects for maintaining 'antisense' accidental expression in order to control expression of genes whose higher dosages may harm the cells. Mechanistically, this is presumably due to the fact that antisense transcription collides with the RNA polymerase that express-es the toxin genes.

Supplementary Figure 6

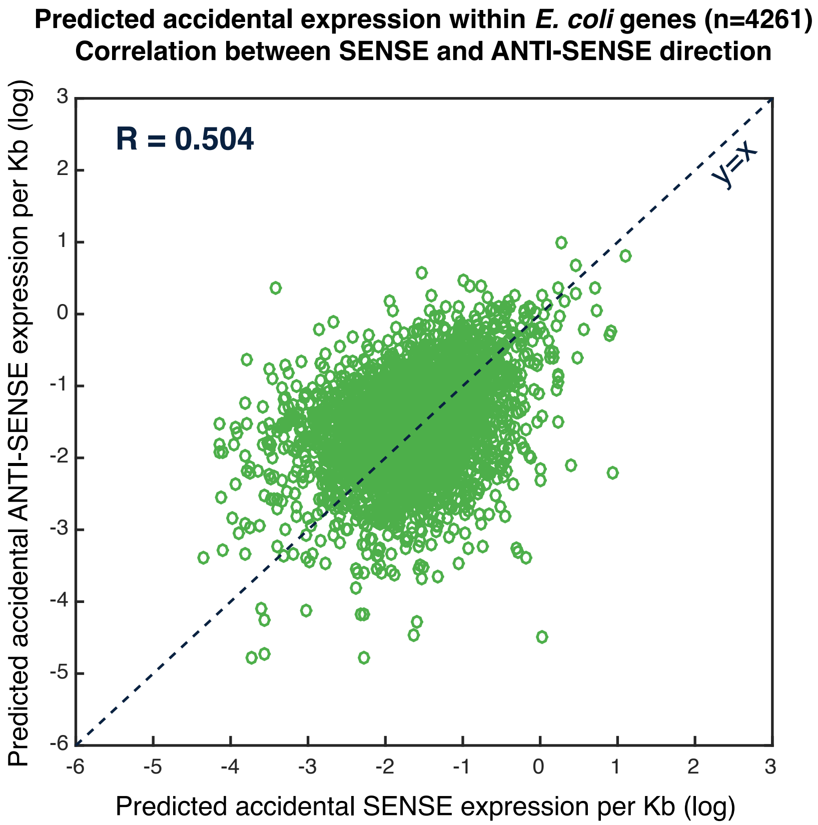

Supplementary Figure 6:

Genome-wide correlation between predicted accidental expression in 'sense' and 'anti-sense' directions - For each WT gene of E. coli we split the score obtained for accidental expression into its two contribut-ing directions (each gene is represented by a green circle). A general correlation (R=0.504) is observed between 'sense' and 'anti-sense' directions.

Supplementary Table 1

List of 20 most depleted six-mers in the WT coding region			
**Six-mer**	**Log2 Fold Change**	**Zscore in Recoded**	**Remarks**
AATAAT	-1.87	-40	-10 motif of 24 WT constitutive genes, for example *yifB*
AGAATG	-1.86	-37	Any base (A/T/G/C) upstream to this six-mer creates a -10 motif found in WT constitutive genes
ATAATA	-2.03	-36	Anti-sense -10 motif of 13 WT constitutive genes, for example *osmB*
ATAATG	-1.90	-40	Anti-sense -10 motif of a WT constitutive gene *yfdV*
GAGAAT	-1.99	-39	-10 motif of a WT constitutive gene *fabI*
GACAAT	-1.55	-34	-10 motif of a WT constitutive gene *yghW*
AGGAGG	-2.80	-36	Ribosome binding site motif
GAGGAG	-2.49	-36	Ribosome binding site motif (http://parts.igem.org/Ribosome_Binding_Sites/Catalog)
GGAGAC	-2.44	-33	Ribosome binding site motif (http://parts.igem.org/Ribosome_Binding_Sites/Prokaryotic/Constitutive/Anderson)
GAGGGA	-2.93	-33	Ribosome binding site motif (http://parts.igem.org/Ribosome_Binding_Sites/Prokaryotic/Constitutive/Community_Collection)
GGAGGG	-2.87	-33	Ribosome binding site motif (http://parts.igem.org/Ribosome_Binding_Sites/Prokaryotic/Constitutive/Anderson)
GGAGGA	-2.33	-35	Ribosome binding site motif (http://parts.igem.org/Ribosome_Binding_Sites/Catalog)
AAGGAG	-2.38	-34	Ribosome binding site motif (http://parts.igem.org/Ribosome_Binding_Sites/Catalog)
GAGGAA	-1.81	-34	Ribosome binding site motif (http://parts.igem.org/Ribosome_Binding_Sites/Catalog)
ATGGAG	-1.51	-33	Ribosome binding site motif (http://parts.igem.org/Ribosome_Binding_Sites/Prokaryotic/Constitutive/Anderson)
CTTGGA	-3.54	-34	Ribosome binding site motif (http://parts.igem.org/Ribosome_Binding_Sites/Prokaryotic/Constitutive/Anderson)
TTGGAG	-3.31	-35	Ribosome binding site motif (http://parts.igem.org/Ribosome_Binding_Sites/Prokaryotic/Constitutive/Anderson)
TTTGGA	-2.79	-33	Ribosome binding site motif (http://parts.igem.org/Ribosome_Binding_Sites/Prokaryotic/Constitutive/Anderson)
TTGGAA	-2.76	-34	Ribosome binding site motif (http://parts.igem.org/Ribosome_Binding_Sites/Prokaryotic/Constitutive/Anderson)
ATAGAC	-3.61	-33	Part of the DNA binding sequence of the *metJ* (represses genes involved in biosynthesis and transport of methionine)

List of 20 most over-represented six-mers in the WT coding region

Six-mer	Log2 Fold Change	Zscore in Recoded	Remarks
**GCTGG**A	+1.77	101	Part of E.coli’s Chi sequence
**GCTGGT**	+1.74	89	Part of E.coli’s Chi sequence
**CTGGTG**	+1.99	103	Part of E.coli’s Chi sequence
**CTGG**CG	+2.32	158	Part of E.coli’s Chi sequence
**CTGCTG**	+1.95	98	Part of E.coli’s Chi sequence
T**GCTGG**	+1.99	124	Part of E.coli’s Chi sequence
GT**GCTG**	+1.97	92	Part of E.coli’s Chi sequence
C**GCTGG**	+2.34	156	Part of E.coli’s Chi sequence
**GCTGG**C	+1.94	119	Part of E.coli’s Chi sequence
GC**GCTG**	+2.21	130	Part of E.coli’s Chi sequence
CC**GCTG**	+1.81	83	Part of E.coli’s Chi sequence
**TGG**CGC	+1.66	82	Part of E.coli’s Chi sequence
**TGATT**G	+3.36	162	Part of consensus sequence of *murR* (DNA binding protein repressing genes involved in catabolism of cell wall sugars)
G**TGATT**	+2.93	110	Part of consensus sequence of *murR* (DNA binding protein repressing genes involved in catabolism of cell wall sugars)
C**TGATT**	+2.75	117	Part of consensus sequence of *murR* (DNA binding protein repressing genes involved in catabolism of cell wall sugars)
**TGATT**A	+2.44	94	Part of consensus sequence of *murR* (DNA binding protein repressing genes involved in catabolism of cell wall sugars)
**GATTG**C	+2.33	87	Part of consensus sequence of *murR* (DNA binding protein repressing genes involved in catabolism of cell wall sugars)
GC**GATT**	+2.13	81	Part of consensus sequence of *cra* DNA binding protein (catabolic repression)
**ATTG**AT	+2.44	85	------------
**ATTG**CC	+2.46	106	------------

Supplementary Table 1:

Counting all six-mers in the coding region of *E. coli* shows depletion of promoter motifs and ribosome-binding sites

We counted occurrences for each possible six-mer in all *E. coli* genes. In addition, we did the same counting but for the 1000 alternative versions we recoded (by encoding the same amino acid sequence and while preserving the overall codon bias). Next, we compared the Z-score for each six-mer representing the rank of the WT count to the 1000 recoded genomes. Shown in the table are the 40 six-mers with the most extreme Z-scores, 20 from each side (20 most depleted six-mers in the upper part of the table, and 20 most over-represented six-mers in the lower part of the table). Overall, promoter motifs (specifically ‘minus-10’) and ribosome binding site motifs are depleted in the coding region of *E. coli*. On the other hand, Chi sequences are over represented in the coding region.

Supplementary Information

Supplementary note 1:

Evolution of de novo promoters on plasmids vs. chromosomal copy –

An experimental concern in the study of de novo promoters is the use of plasmids vs. a chromosomal copy. Measuring expression from a promoter that evolves on plasmids is highly affected by the copy number of the plasmid and higher expression can be achieved by mutations that increase the plasmid copy number, rather than by actual promoter mutations. In such cases very small expression signals can yield an overall significant expression merely due to multiple copies, compared to effective promoter mutations that strongly increase expression of a single copy on the chromosome. Therefore, our setup used a single chromosomal copy as a starting point for evolving promoters.

Supplementary note 2:

Expression activation by capturing an existing promoter or a mutation in the intergenic region upstream to the random sequence – For the random sequences listed in Supplementary Data 1 as evolved by capturing an existing promoter upstream, we observed various deletions in the intergenic region upstream to the lac genes. All of these deletions placed the lac genes in front of the upstream chloramphenicol selection gene. These deletions also eliminated the termination sequences that separated the lac genes from the genes upstream.

In strains whose activating mutations appeared in the intergenic region, but upstream to the random sequence, de novo promoters were detected in some cases by mutations that are similar to the mutations which created de novo promoters in the random sequences (detailed in the mutations table). Yet, there was a group of point mutations, all at the same nucleotide, that did not seem to create a promoter by creating a promoter motif, or to affect a potential transcription factor binding site of a nearby predicted promoter. Nevertheless, each one of these mutations was sufficient for expression of the lac genes. The mutated sequence in the intergenic region was tcgaaagactgggcctttcgttttat, where the 'g' in the middle of this sequence was mutated multiple times in different strains. In some cases from g to T, in other cases from g to A and once the g was deleted (1 base deletion). It is unclear what was the mechanism by which these mutations activated expression.

We hypothesize that random sequences whose expression evolved via mutations in the intergenic region could not find an activating mutation in the random sequence. For such sequences, a mutation in the random sequence that can induce expression might not exist. Therefore, we took such a sequence, RandSeq27, and computed mutations that might improve its chances of becoming an active promoter. To this end, we scanned the original RandSeq27 for maximal matches to the canonical promoter. Since there were multiple matches, we chose the maximal match with an optimal spacer of 17 bases. Then, we introduce a point mutation that improved the minus-10 motif. After introducing this mutation into RandSeq27, it did not show promoter activity, yet after applying selection for growth on lactose (like in the library of ransom sequences)

the strain found a second mutation that together with the first one we inserted exhibited expression of the lac genes:

RandSeq27 – inactive promoter:

```
cggtccgtttataacatgcgcagaggaagctgtctgtgcgtcgccagactcagagacccttatactacacccgcctggctgcgaatcatccaccactttaagt
```

RandSeq27 + 1st mutation (computed) – still inactive promoter:

```
cggtccgtttataacatgcgcagaggaagctgtctgtgcgtcgccagactcagagacccttatactacacccgcctggctgcgTatcatccaccactttaagt
```

RandSeq27 + 2nd mutation (via selection) – active promoter:

```
cggtccgtttataacatgcgcagaggaagctgtctgtgcgtcgccagactcagagaccctt-tactacacccgcctggctgcgTatcatccaccactttaagt
                                                           TTtACt--------17--------TATcAT
```

This might imply that such sequences are two mutations away from functioning as active promoters.

Supplementary note 3:

Position weight matrix scores of WT constitutive promoters do not account for transcription factors

Our results showed that the majority of ~100bp random sequences get promoter scores similar to those of WT constitutive promoters with only one mutation. Nonetheless, one should bear in mind that constitutive WT promoters may also utilize additional motifs and transcription activators that may express them to higher levels than our evolved promoters. These additional motifs and transcription activators are not reflected in the promoter score calculated according to the position-specific matrix. Therefore, our experimental result that active promoters evolve from random sequences by capturing the canonical motifs emphasize that newly evolved promoters can get substantial activity even without additional transaction activators.

Supplementary note 4:

Estimating accidental expression by BPROM rather than by scan with position weight matrix

In our experimental library none of the activating mutations created/improved other promoter motifs rather than the -10 and the -35, like the UP element or the TGn motif. Nonetheless, when we were to estimate putative accidental expression from the *E. coli* genome we aimed for a software that takes into account all known promoter elements and not only the two major ones. Therefore, a programed scan of the genome that factors in all features that affect expression seemed like the optimal approach. Furthermore, since such software dedicated for this purpose are already available we though it would be better to use them rather than to develop one in-house. We chose BPROM merely because it was the only software, which the developers allowed us batch runs from a script (for other software we could only run limited amount of queries and on an a user-required website and not via scripts).

Supplementary note 5:

Using an alternative model for recoding of the *E. coli*'s coding sequences

Our first recoding model represent the standard recoding method that requires the recoded versions to encode the exact same amino-acid sequence as well as to comply with the codon bias. This recoding model showed that the wild-type version is depleted from accidental promoters, which means that the depletion observed could not stem from a confounding effect of the amino-acid sequence or the codon bias. Nonetheless, other cofounding affect might have caused the results that showed minimization of accidental expression. Due to this concern, we looked for alternative recoding models that can rule out potential confounding factors other than the ones dismissed by the original recoding model.

Since *E. coli*'s promoter motifs are AT-rich (the two main consensus elements are 10/12 AT, and the additional up elements are either 6/9 or 11/11 AT) we thought that the detected depletion signal was merely due to the fact that when we recode a gene a thousand times (while preserving amino acid sequence and codon bias) some recoded versions will have AT content that deviates from the original AT content of the WT version of the gene. This may lead to more promoter motifs in these recoded versions, and eventually may seem like the WT version is depleted of promoters, while the truth is that some of the recoded version are enriched for promoters due to the AT content bias.

To rule out this option we redid the analysis using another recoding model, this time each recoded version preserved the exact AT content of the WT original version. The recoded versions this time were obtained by reshuffling the original codons of each gene in a thousand different orders. Reassuringly, the results for this recoding method also showed that the WT version is depleted of promoters, indicating that an AT bias is not a confounding factor either (see Supplementary Fig. 4). All together we ruled out, amino-acid sequence biases, codon-biases and AT content biases from being confounding effects that might mislead us to thing that the WT coding region is indeed depleted from promotes. In principle there might be other factors that can have a confounding effect, yet at this point we do not have a concrete additional recoding model to test.

Supplementary note 6:

The different costs of accidental expression and the motivation to focus on toxin-antitoxin gene couples – Accidental expression has a global cost due to waste of resources and occupying cellular machineries. In addition there is also a cost that is due to interference of specific genes. We observed that depletion of accidental expression is more emphasized in essential genes and is less observed in foreign genes like toxin and antitoxin prophage genes. Besides the stronger selective pressure to mitigate interference in essential genes, additional possible reasons for these differences may include: (a) foreign genes have been in the *E. coli* genome for shorter time and thus their expected optimization level is lower, and (b) foreign genes may have lower GC content than *E. coli*, which may affect accidental expression[1] as promoter motifs are AT-rich. To decipher between these potential factors, we therefore focused on toxin/anti-toxin gene couples[2], as for each couple the age in the *E. coli* genome is presumably the same, and

they have similar GC content. Nonetheless, the anti-toxin gene is more important to the *E. coli* fitness than its toxin counterpart. Indeed, we observed lower accidental expression in anti-toxin genes compared with toxin genes. This result implies that for each gene the level of avoiding accidental expression is mainly dependent on how important to the fitness it is to have this gene expressed without interference.

Supplementary note 7:

Toxin Anti-toxin couples – When analyzing toxin-antitoxin gene couples for potential differences in their accidental expression, especially between sense and anti-sense orientations, we excluded gene couples whose orientation in the genome could not lead us to meaningful conclusions. Specifically, we excluded gene couples for the following reasons:

a) Toxin and antitoxin genes were overlapping, hence internal expression affects both (e.g. *ibsA* nad *sibA*).

b) Couples that had this orientation Antitoxin➔ Toxin➔ in which antisense expression from within the toxin gene also influences the adjacent upstream antitoxin (e.g. *yafQ* and *dinJ*).

c) Couples where the annotated promoter of the antitoxin gene is within the toxin gene and thus interference to the toxin is from a canonical functional promoter (e.g. *symE* and *symR*).

Supplementary References:

1. Lamberte, L. E. *et al.* Horizontally acquired AT-rich genes in Escherichia coli cause toxicity by sequestering RNA polymerase. *Nat. Microbiol.* **2,** 16249 (2017).
2. Keseler, I. M. *et al.* EcoCyc: fusing model organism databases with systems biology. *Nucleic Acids Res.* **41,** D605-12 (2013).
