# Supplementary material for: Random sequences rapidly evolve into de novo promoters
Source: Nat Commun. 2018 Apr 18;9:1530. doi: 10.1038/s41467-018-04026-w (PMC5906472; doi:10.1038/s41467-018-04026-w)
Supplement: Supplementary file 3 — Description of Additional Supplementary File [file 41467_2018_4026_MOESM3_ESM.pdf]

Description of Additional Supplementary File

File Name: Supplementary Data 1
Description: A list of all mutations found in the different replicates of each of the 40 evolving random sequences
